# Bioinorganic Nanoparticles for the Remediation of Environmental Pollution: Critical Appraisal and Potential Avenues

**DOI:** 10.1155/2023/2409642

**Published:** 2023-04-10

**Authors:** Md. Mominur Rahman, Limon Ahmed, Fazilatunnesa Anika, Anha Akter Riya, Sumaiya Khatun Kali, Abdur Rauf, Rohit Sharma

**Affiliations:** ^1^Department of Pharmacy, Faculty of Allied Health Sciences, Daffodil International University, Dhaka 1207, Bangladesh; ^2^Department of Pharmacy, East-West University, Aftabnagar, Dhaka 1212, Bangladesh; ^3^Department of Chemistry, University of Swabi, Swabi, Anbar, KPK, Pakistan; ^4^Department of Rasa Shastra and Bhaishajya Kalpana, Faculty of Ayurveda, Institute of Medical Sciences, Banaras Hindu University, Varanasi 221005, Uttar Pradesh, India

## Abstract

Nowadays, environmental pollution has become a critical issue for both developed and developing countries. Because of excessive industrialization, burning of fossil fuels, mining and exploration, extensive agricultural activities, and plastics, the environment is being contaminated rapidly through soil, air, and water. There are a variety of approaches for treating environmental toxins, but each has its own set of restrictions. As a result, various therapies are accessible, and approaches that are effective, long-lasting, less harmful, and have a superior outcome are extensively demanded. Modern research advances focus more on polymer-based nanoparticles, which are frequently used in drug design, drug delivery systems, environmental remediation, power storage, transformations, and other fields. Bioinorganic nanomaterials could be a better candidate to control contaminants in the environment. In this article, we focused on their synthesis, characterization, photocatalytic process, and contributions to environmental remediation against numerous ecological hazards. In this review article, we also tried to explore their recent advancements and futuristic contributions to control and prevent various pollutants in the environment.

## 1. Introduction

The present world is on the verge of a massive environmental disaster that will ruin us for our fate. The current state of the ecosystem is degrading at an exponential rate. The environmental problems are accumulating worldwide, and we must act as though our planet is urgent. We must adopt a new mindset and face disasters with fresh ideas, solutions, and complete consciousness, and seriousness. Emissions from industrial activities and automobile exhaust are the leading sources of the rest of environmental pollution [1].

Nanomaterials have flourished as a new-fashioned model component in recent times [2, 3]. Taniguchi coined the word “nanotechnology” in 1974 to define the field that works with the manufacture and utilization of nanoscale components (100 nm) [4, 5]. “According to the National nanotechnology initiative (NNI) in the US,” nanotechnology is defined as a field of science, engineering, and technology where materials are practiced at the nanoscale size (1–100 nm), using unique phenomena in a wide range of biology, physics, chemistry, medicine, electronics, and engineering fields [6–8].

It is the newest technology applied in several areas such as physics, chemistry, biology, and recognizing machines [9, 10]. In this regard, nanotechnology might be viewed as a critical platform for reorganizing the different methodologies and techniques used to solve current and future problems such as detectors, sensors, and other significant environmental challenges pollution control and prevention and remediation. The range of nanometres is noteworthy because it closely reflects the size of human proteins. This makes the connection between nanometal research and biology and biochemistry clear. Metal nanoparticles have a lot of exciting features, and it's only a matter of time until more of them are used. The characteristics of molecules change dramatically when they shrink. The new electronic form has redefined several outstanding features as electronic, optical, magnetic, reactive, adsorbent, and mechanical [11].

Nanomaterials were the most robust materials due to their numerous structures and forms, like as nanowires, nanorods, nano-branches, nano-spheres, nano-cubes, nano-bipyramids, nano-flowers, nano-cages and nano-shells [12]. Nanotechnology and its products, known as nanomaterials, are being applied in numerous sectors, including healthcare, industry, electronics, cosmetics, pharmacology, bio clinical, and biological fields, among others [13, 14].

Nanocomposites and nanostructured materials are two types of nanomaterials. They are employed very successfully and effectively in different areas and they are also particularly dynamic because of their tiny size with much exterior space. As a result, they can be used in various ways as toxicants, absorbents, and adsorbents for pollutants [5]. In the last couple of decades, different physical and chemical processes have been promoted for generating nanoparticles of diverse sizes, shapes, and forms in response to the rising need for several nanomaterials of metal and nonmetal [15]. Researchers have lately started investigating numerous nanomaterials functionalized substances to apply in water and environmental treatment, such as the rosary, nanowires, membranes, foams, and so on, which are based mainly on polymeric materials [16–18].

Nanoscience has the potential to make a significant contribution to the development of pure and emphatic technologies that have outstanding advantages both in health and environment [19]. It is much compatible for update versions of technologies. Several nano-based techniques are being studied to improve the efficiency of essential sustainability clean-up operations and provide environmental damage management and mitigation solutions [20]. Nanoscience can lower the power consumption in several constructions and thus assist the environment. Also, it will produce goods that will be able to regenerate after use, promoting and deploying eco-friendly components [21].

The aim of this study is to reveal and discuss various types of potential synthesis and characterization of bioinorganic nanoparticles along with their spanned applications against environmental hazards.

## 2. Environmental Pollution and Pollutants

Pollution of the environment is a global issue that can considerably influence human health. Poverty, weak legislation, and a lack of awareness of pollution contribute to higher pollution levels in medium and low-income countries than in developed countries [22]. The repercussions are felt by humans and other aquatic and terrestrial creatures, including microorganisms, who can maintain the biogeochemical activities required for ecosystem life due to their abundance and diversity.

Toxins are particles that harm the environment by polluting it. Pollutants enter the atmosphere in various methods, both naturally and as a result of human activity [23]. The dyes, heavy metals, insecticides, and polyaromatic hydrocarbons are examples of pollutants [24].

### 2.1. Heavy Metals

Heavy metals are categorized because of significantly higher molecular weights or densities. The phrase “heavy metal” is increasingly used to describe environmentally and human harmful metallic chemical components and metalloids [25]. The Fe, Cd, Ni, Ti, Ag, Zn, Cr, Mn, Co, Pt, Au, As, Hg, Sn, and Pb are numerous heavy metals [26, 27].

### 2.2. Dyes

Many types of organic synthetic chemicals are used in diverse industrial operations, called dyes. Consequently, they have turned into general industrial environmental pollutants throughout their several productions [28]. One persistent organic pollutants (POPs) example is colorants, and the fact that they are xenobiotic has been a source of worry [29]. Both persons and environments are being affected by this. Mutagenic pigments distribute red 1 and distribute orange 1 are present in nature. Sudan I dye (now prohibited), basic red 9 color (which causes cancers in fishes), and crystal violet color (which creates malignancies in humans) are all harmful [30].

### 2.3. Pesticides

Pesticides are chemicals used in fields, crops, and other public places to destroy undesired organisms. Pesticides have long been used to protect crops and livestock against bug infestations and reduce output losses. Pesticides include insecticides (organophosphates), herbicides (paraquat and diquat), and fungicides (di-thiocarbamates). Pesticides have long been used to protect crops and livestock against bug infestations and reduce output losses. Pesticides include insecticides (organophosphates), herbicides (paraquat and diquat), and fungicides (di-thiocarbamates) [31]. Despite their widespread use, pesticides may pose a health hazard to the ecosystem and all living things [27, 32–35].

### 2.4. Polyaromatic Hydrocarbon

One of the global environmental pollutants is a polyaromatic hydrocarbon (PAH), a group of persistent and that is poisonous, mutagenic, and carcinogenic to humans. Demand for petroleum goods has increased, which has increased emissions. One aspect is the combustion of organic products such as coal and firewood in the lack of oxygen [23, 36, 37].

## 3. Impact of Pollutants on Environment and Humans

### 3.1. On Environment

As a critical source of life on earth, water contamination has attracted public interest and is one of the most vulnerable environmental compartments [38]. One of the most prominent forms of aquatic pollution is sociocultural eutrophication caused by environmental toxins [39]. Ocean acidification results from increased atmospheric CO_2_ and traces element levels in the ocean, altering organisms' acid-base equilibrium, decreasing their survival chances and performance. It has caused coral reefs, microorganisms like zooplankton, and shellfish to calcify at the wrong time [40]. Various pollutants, including aromatics, plasticizers, and numerous xenobiotic chemicals, are unlawfully deposited, posing a threat to the terrestrial ecology [41].

Air pollution is the world's sixth-leading cause of death, posing a threat to human civilization. Different types of hazardous chemicals like halocarbons, CO_2_, N_2_O methane, and ozone are only a few examples of air pollutants contributing to one of the most critical environmental issues for global warming [42].

### 3.2. On Humans

Environmental pollution has such a negative impact on human health that it has been linked to nearly all human diseases [43]. A collection of contaminants in the air produces oxidative stress and raises cancer risk [44]. The most common health problem is sensitivity to air contaminants, including oxides, fine particles, and ground-level ozone. CO causes a shortage of O_2_ supply to different parts, impairs eyesight, and can be lethal in high doses. Sulfur oxides are carcinogenic when exposed to them for an extended period [45].

Nitrogenous pollutants and fine and ultrafine particles conduce vaso-control. Also, they may cause synaptic inflammation as they pass through the blood-brain barrier (BBB) and reach the brain, therefore, paving the way for future neuro dysregulations [15, 46–61]. Pesticides, chemically generated fertilizers, dyes, detergents, and heavy metals are all making their way into aquatic systems, posing a threat to human health [62].

## 4. Nanotechnology and Nanomaterials

Nanotechnology is a cutting-edge technology with biology, sensing, medicine, chemistry, and physics [9, 63]. Nanoparticles are the most crucial feature of nanotechnology: tiny molecules with a size of one-to-one hundred nm. They are composed of metals, metal oxide, carbon, or organic molecules [64].

Inorganic nanoparticles are comparatively biocompatible, hydrophilic, and non-toxic, highly stable than organic particles [65]. In response to different structures, nanoparticles have a large diversity of shapes, dimensions, and sizes [66]. Nanoparticles can be one dimension on graphene, two-dimension like carbon nanofibers, or three-dimension, containing all three dimensions, for example, gold nanoparticles. When it was found that a particular size gets into a particle's physicochemical properties and optical efficacy, nanomaterials' worth was raised. There are three layers to NPs to functionalize the nanomaterials in the surface layer. Various biomolecules, surfactants, trace metals, and polymers are used. In contrast to the core, the second layer is a shell layer of numerous chemical components. The essential element of the NP is called the core, and it usually refers to the NP itself [67, 68].

Nanoparticles are employed for various purposes, from medicinal treatments to diverse sectors, such as sunlight and atomic fuel electrodes for energy production, to everyday things like cosmetics and clothes [69].

## 5. Classification of Inorganic Nanoparticles

In comparison to organic materials, nanomaterials are non-toxic, hydrophilic, compatible, and highly stable. Below is a list of the numerous types of inorganic nanoparticles.

### 5.1. Metal-Based Inorganic Nanoparticles

Metal-based nanocrystals with sizes varying from 1 to 100 nanometres, a broad exterior ratio with mass, charge density, noncrystalline and crystals frameworks, globular or tubular, vibrant, high sensitivity, and responsivity to humidity, air, thermal, and sunlight are all characteristics of metal-based NPs [70]. Metals such as iron (Fe), silver (Ag), aluminium (Al), cadmium (Cd), copper (Cu), gold (Au), cobalt (Co), lead (Pb), and zinc (Zn) are used to make metal-based NPs [71]. Because they can be used to create medicines, these nanoparticles have attracted much attention in the pharmaceutical industry [72].

### 5.2. Metal Oxide-Based Nanoparticles

Metal-oxide-based NPs can be used in food production and ecological sustainability, and they can be made cheaply with readily available ingredients. In comparison to their metal counterparts, these NPs exhibit superior characteristics. The TiO_2_, ZnO, Ag_2_O, CuO, FeO, MnO_2_, Al_2_O_3_, Bi_2_O_3_, MgO, and CaO are metal oxide-based NPS discovered to boost their activity and have substantial antibacterial capabilities [73].

### 5.3. Doped Metal/Metal-Based Nanoparticles (NPs)

Chemical alterations to NPs can be done to create more stable molecules that are also environmentally friendly. Photo electrochemical and bactericidal properties were found in new ZnO nanoparticles. Through analyzing the structural conditions and optical characteristics of doped Mn/ZnO nanoparticles uncovered that those doped nanomaterials exhibited increased efficacy [74]. Ag-doped MgO in biomedical inflictions performed as a better antimicrobial agent than pure Mg oxides over *S. aureus* [75–77]. As a result, NPs based on doped metal oxides beat pure oxides in antibacterial activity [78].

### 5.4. Metal Sulfide-Based Nanoparticles

Chemical procedures were used to amalgamate semiconductor materials sulfide NPs into polymers to preserve their surfaces [79]. Poly-methyl methacrylate (PMMA) has become the most fully observed polymer among many accessible polymers due to its substantial chemicophysical and mechanical qualities [80]. Metallic sulfides containing the conjugated polymers sulfur have been studied and shown to be a necessary toxic-free metal, piquing biomedical interest [81].

## 6. Synthesis of Nanoparticles

**Figure 1:**
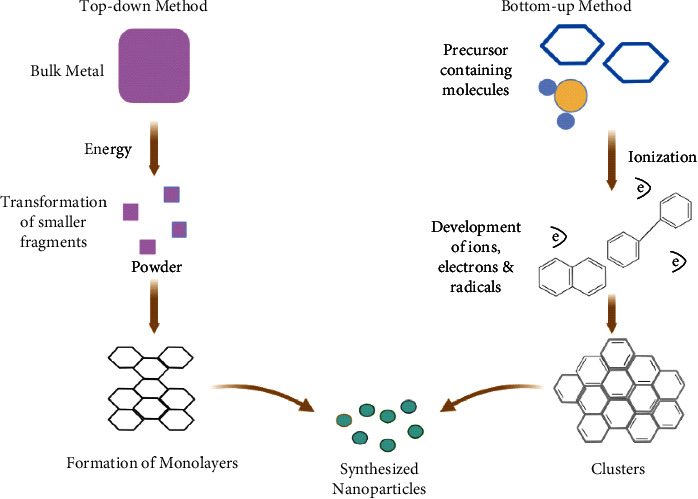
Schematic presentation of top-down and bottom-up methods of carbon-based nanomaterials.

Diverse ways of synthetic approaches have been applied to produce new and more effective nanomaterials. Synthesis of nanomaterials can be done in various ways. Top-down and bottom-up techniques are the two main categories (Figure 1).

### 6.1. Top-Down Method

A bulk substance is reduced to nanoscale-level particles via a highest or destructive approach (Figure 1). Mechanical size reduction nanotechnology, laser irradiation blasting, and temperature breakdown are the operations that follow this approach. Mechanical mills are the most widely employed among the various top-down ways to create different nanoparticles. Whenever various aspects are processed in an inert environment at the time of nanoparticles production, an instrumental milling technique is used to operate milling and postannealing [82]. Nanolithography refers to the study of making a specific range of structured nanoparticles with at least one-dimensional in a size range of one-to-one hundred nm [83]. The ability to construct anything other than individual particles into a group with the right shape and size is one of the nanolithography's key features [84]. A common approach for synthesizing nanoparticles from various liquids is LASiS (laser ablation synthesis in solution) [85].

### 6.2. Bottom-Up Method

From particles to clusters to nanoparticles, the upper or proactive process accumulates material (Figure 1). The most extensively utilized nanoparticle synthesis methods are sol-gel, rotating, biosynthesis, chemical vapor deposition (CVD), and decomposition. The majority of the nanoparticles are made by the sol-gel method because of their significant ability with simplicity. That is why it is the most popular bottom-up technique [86]. Pyrolysis is the most extensively used technology for manufacturing nanoparticles on a large scale. It entails using a flame to ignite a precursor [87]. Pyrolysis is a practical, simple, inexpensive, and continual process that produces a large yield [88]. Green synthesis of biosynthesis has proven to be the most beneficial [89]. Synthesis of nanoparticles is an environmental friend and an acceptable technique for generating biodegradable and nontoxic nanoparticles [88].

### 6.3. Microorganisms-Based Inorganic Nanoparticles Synthesis

Together with prokaryotes and eukaryotes, a variety of microorganisms produce platinum, cadmium, silver, iron, gold, zirconium, palladium, and metal oxides (inorganic nanoparticles) as ZnO, TiO_2_, and others. Bacteria, actinomycetes, fungi, and algae are among these microorganisms. Nanoparticles can be generated intracellularly or extracellularly, depending on their location [90, 91]. Many metals that have been employed in other professions, such as medicine are used in green synthesis [92].

Production of nanomaterials can be performed accurately and quantitatively using either an external or intracellular technique, depending on the features of algae. The polysaccharides, minimizing carbohydrates, proteins, peptides, and other reducing compounds have been proposed as potential external metallic nanoparticle makers, precipitating minimizing metal ions to nanoparticles [93]. Metal ions are reduced in algae by photosynthesis and respiration, resulting in the creation of metallic nanoparticles inside the cells.

Engaging in a photorespiration electron transport system (PETS) and a respiratory electron transport system (ETS) discovered on the cellular membranes and in the cytoplasm of algae was reported as the synthesis mechanism [94]. Different nanoparticles synthesized by various microorganisms are listed in the table below (Table 1).

Helping in extracting from the red *Macroalga gracilaria* targets was used to investigate the extracellular production of ZnO NPs (S. G. Gmelin). A change in color from white to pale brown can be seen during electron transfer of zinc ions (Zn^2+^) utilizing zinc nitrate (Zn (NO_3_)_2_) as a metal catalyst, indicating the creation of ZnO NPs. Rod-shaped nanoparticles with sizes varying from 66–95 nanometres were generated by the researchers [106].

*Anabaena doliolum*, a nitrogen-fixing cyanobacterium, has been used to biosynthesize Ag NPs. The hue of the Ag^+^ methods from reddish-blue to dark brown during bio reduction indicates the production of Ag NPs [107].

*Fusarium oxysporum*, a fungus, has been used in several investigations to create metallic nanoparticles, mainly silver (Ag) nanoparticles. Pure Ag NPs in size vary between 5 and 15 nanometres were generated, and it was proposed that proteins secreted by the fungus stabilized them. Although the revelation that *F. oxysporum* formed metal nanoparticles extracellularly, rather than the intracellular generation of Ag and Au NPs reported in previous investigations, was the most significant advancement in the fungal synthesis of metal nanomaterials [108].

According to the mainstream consensus, bacteria can create metal and metalloid NPs both intracellularly and extracellularly. In an extracellular process, proteins, enzymes, and biomolecules in the medium and cell wall components decrease ions. Extracellular reduction seems to favor intracellular reduction since it is cheap, simpler to extract, and more efficient. However, electrostatic interactions connect iron and metals ions to functional groups on the cell wall in the intracellular process. After entering the cells, the ions combine with intracellular proteins and cofactors to form NPs [109].

## 7. Characterization of Bioinorganic Nanomaterials

**Figure 2:**
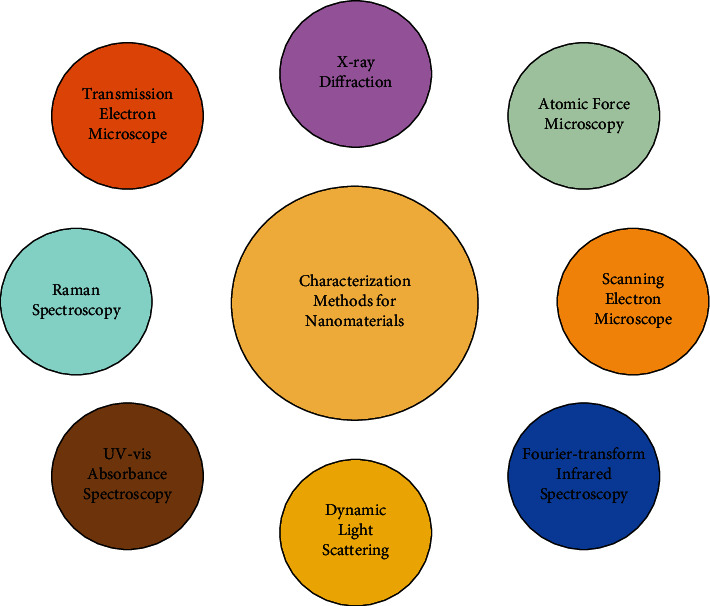
Different characterization methods for nanomaterials [24].

Several techniques have been used to characterize the size, crystal structure, elemental composition, and a variety of other physical properties of nanoparticles (Figure 2 and Table 2). In several cases, there are physical properties that can be evaluated by more than one technique. These techniques have some potential advantages over the other techniques for characterization of nanomaterials such as they are highly sensitive, less restrictive, require only a small volume of sample, linearity over a wide range of concentration, more efficient, rapid and powerful technique, cost-effective, and also widely available. UV-vis spectroscopy is one of them, which works based on the principle of surface plaque resonance (SPR) [110]. Scanning electron microscopy (SEM), transmission electron microscopy (TEM), and atomic force microscopy (AFM), all of these techniques work with the parameter of surface charge, size, and morphology. Another method, named Fourier-transform infrared (FT-IR), is used to determine functional groups [111]. To identify the crystalline phase, the X-ray diffraction (XRD) method has been used [112–114]. Nanoparticles size measurement uses a dynamic light scattering process [24].

### 7.1. Na-TiNT Nanocrystals

#### 7.1.1. Raman Spectroscopy

The presence of Na-TiNTs in a powder sample was investigated using Raman spectroscopy. A Raman spectrometer was created by connecting a stereoscopic optical microscope lens to a Raman spectrometer. Experiments were conducted using an argon (Ar) laser, a greenish laser with a wavelength of approximately 532 nanometres, and a Gaussian sector distribution with a maximum power output of megawatts that could be modified. The spectra were acquired in one second with a laser power of 300 W; by using such low power, it was able to avoid burning the sample and capturing an optical gesture, and a scanning region of 30 m × 30 m was created in the powder sample for the photometric image [130].

#### 7.1.2. Diffraction or Light Scattering of X-Rays

A Bruker diffractometer, model D8 advance, was used to measure X-ray diffraction on a copper ampoule 0.15406 nanometres with an angular variation (2) of 5° –70°, a step of 0.02°, and current and voltage of 40 mA and 40 kV, respectively. These procedures were carried out at the Federal University of Ciara's Department of Engineering and Material Sciences [130].

#### 7.1.3. Transmission Electron Microscopy (TEM) and Scanning Electron Microscopy (SEM)

Scanning electron microscopy (SEM) and energy dispersive X-ray analyzer (EDX) data were taken with a Quanta FEG 50 field emission microscope with a 10 mm work distance. In comparison, transmission electron microscopy (TEM) was done with a Zeiss LIBRA 120 electronic microscope [130].

#### 7.1.4. Pathogenic Substances and Varieties

In this experiment, Gram-positive and Gram-negative strains were employed, which were cultured at the Universidad Regional do Cariri's Laboratory of Microbiology and Molecular Biology (LMBM) (URCA). At least one of the antibiotic classes employed is resistant to each strain used. All of the chemicals utilized with antibiotics and products were diluted to 1024 g/ml in sterile water. The highest limits for this chemical were observed in drugs that required the previous dilution in DMSO, as per the Clinical and Laboratory Standards Institute [130].

#### 7.1.5. Antibacterial Effectiveness and Drug Modulation Assays

The broth microdilution technique determined MIC in immediate anti-bacterial activity and the altering impact of the Na-TiNT antibiotic activity. In all experiments, turbidity was evaluated utilizing the McFarland scale 0.5 (1 108 CFU/mL), and inoculants were produced from twenty-four-hour- colonies of pathogenic bacteria in HIA (Heart Infusion Agar) adjusted in 0.9 percent saline. This approach proposed by Javadpour and co-workers was utilized to test direct anti-bacterial activity [131].

A solution consisting of 10 percent Brain Heart Infusion broth and 10 percent bacterial swab was put onto the microdilution plates. The wells were then micro-diluted with Na-TiNT at concentrations of 512 to 8 g/ml. The antibiotic-altering function test followed the methods published by Coutinho and colleagues [132]. The disc wells were loaded with a solution similar to that used in the direct efficacy test, adding a sub-inhibitory concentration of Na-TiNT. Antibiotics were then micro diluted over the plates in concentrations ranging from 512 g/ml to 0.5 g/ml. The two experiments were carried out in triplicate, with colorimetric readings using 20 mL of reassuring in each well following a 24-hour incubation at 37°C [130].

#### 7.1.6. Statistical Analysis

The geometrical methods of the three replications have been used as central data in the standard error of the mean analysis. The evaluation has been carried out using GraphPad Prism 5.0, which includes two different way ANOVA followed by a Bonferroni post hoc test. *P* 0.05 values are taken significantly [130].

### 7.2. Nano CaCO_3_

For the manufacture of nano CaCO_3_, the elegant approach of in situ deposition technique was adopted. The nanosize of the granules was confirmed using the diffraction of the X-ray technique. The enthalpy was calculated using differential scanning calorimetry (DSC). The nano CaCO_3_ polypropylene (PP) composites were made by combining 2 and 10% of different CaCO_3_ nanosized (21–39 nm) in a polypropylene (PP) matrix. In the case of 2 wt. percent of a 30 nm sized quantity of CaCO_3_ in a PP composite, phase conversion was observed. For six wt. percent nano CaCO_3_, a decrease in nanosize from 39–21 nm resulted in a reduction of H and percent crystallinity and increased melt temperature. The tensile strength increased as nano CaCO_3_ increased, with the smaller particle size showing the most significant improvement. The production of a greater number of tiny spherulites uniformly present in the PP matrix is responsible for improving thermal and mechanical properties [133].

### 7.3. Nano Fe_3_O_4_

A two-step nano-emulsion technique was employed in this study to generate nano-Fe_3_O_4_ particles. The molar ratio of liquid to NP-5 (R), alkali level, temp, total fixed ferrous content, and growing interval were all factors that could impact the form and nanostructure. It was discovered that such generated nano-magnetite particles had uniform size and good shape with *R* = 6.0, alkali level = 2.5 mole/L, temp = 30°C, and initial total ferrous level = 1.75 mole/L. Ostwald ripening and long aging durations can form cubic Fe_3_O_4_ nano-powder with 100–400 nm. The crystal structure of the generated nanoparticle is related to the cubic system, as determined by selected area electron scattering (SAED) and indexed by the X-ray diffraction (XRD) pattern [134].

### 7.4. Nano TiO_2_

#### 7.4.1. UV-Vis Spectrum

UV-Vis spectra is a powerful analytical chemistry technique for assessing the photocatalytic activity of titanium dioxide nanoparticles in polar solvents [135].

In UV-Vis's spectroscopy, TiO_2_ absorption appears at 280 nm. Roopa et al. [136] reported on similar research as well. As per Vijayalakshmi and Rajendran, the spectra exhibit a spectral range of 200–1200 nm, with a peak at 354 nm wavelength and intensity of 0.86, indicating considerable UV absorbance [137].

The UV-Vis absorbance spectrum of TiO_2_ NPs biogenically produced from Aloe vera extract indicated an absorption coefficient of 3.503 eV. The absorption bands of nanocomposite reveal a strong sensitivity below 400 nm. The absorbance peak of the TiO_2_ material oxidized around 400 degrees celsius is about 393 nanometres, with an energy gap of 3.196 eV that matches the crystalline phase bandgap perfectly [138].

#### 7.4.2. Thermogravimetric Analysis

Thermogravimetric analysis (TGA), a technology in which the mass of a sample is measured versus time or temperature, is widely used in polymer research, especially to investigate the thermal stability of polymeric systems under real-world environments. TGA gives quantifiable data through curve on the weight loss or gain process. The thermal stability of TiO_2_ with polycarbonate was demonstrated using TGA. A Perkin-Elmer system was used to conduct TGA. At a heating rate of 10°C/min and a nitrogen flow of 70 mL/min, the temperature range was changed from room temperature to 800°C. A BUEHLER (model 60044 USA) microhardness tester with a mounted microscope was used to conduct Vickers microhardness testing. TGA curves reveal that TiO_2_ nanocomposite films have stronger thermal resistance than polycarbonate films, and this resistance appears to grow as the TiO_2_ concentration rises [139].

#### 7.4.3. Characterized by Using SEM

TiO_2_ NPs were produced by Aeromonas hydrophilic bacteria. The FESEM analysis approach was also used to explore the surface of nanoparticles. The formation of aggregated nanoparticles revealed that the nanoparticles were evenly distributed on the surface.

It demonstrates that particles were tightly scattered over a small dispersion range [140]. Nanoparticles with a smooth surface morphology were used [137].

#### 7.4.4. Characterized by Using TEM

Transmission electron spectroscopy (TEM) examination revealed the shape, and structural arrangement of bio-template aided synthesized TiO_2_ NPs. This investigation could be utilized to figure out the properties of the nanomaterials that are being formed and learn further about them. Trigonella foenum graecum-assisted TiO_2_ NPs had an average particle size of 20 nanometres, and HR-TEM images revealed spherical-shaped polydisperse nanoparticles. As demonstrated in the TEM image, the individual nanoparticles were nearly uniform, with a range of 40–60 nanometres [141].

This research shows that using the biogenic technique, small-sized and effective anatase TiO_2_ nanoparticles may be obtained for the necessary applications. *d* = 0.357 nanometres was calculated as the determined *d*-spacing value, which is extremely similar to the XRD *d*-spacing value [142].

#### 7.4.5. Characterized by Using FT-IR Spectrum

The Fourier-transform infrared spectroscopy estimation technique could give the needed information regarding biologically active ingredients/molecules from natural goods that operate as a template throughout the synthesis process to keep TiO_2_ NPs avoid overgrowing, aggregating, and losing phase stability. In the FT-IR spectrum, the surface water and hydroxyl groups were detected at 3430 and 1643 cm^1^, respectively. The spectrum intensities of the spectra for the generated TiO_2_ NPs are 3430, 2937, 1643, 1403, and 1079 cm^1^. These findings demonstrated that *A. hydrophilia* contained solvents, phenolic compounds, primary amines, lactones, and aliphatic amines, most of which might have been engaged in the synthesis process and thus retain phase stability [143].

## 8. Production and Utilization of Carbon-Based Nanomaterials

### 8.1. Carbon Nanoparticles with Low Dimensions

#### 8.1.1. Carbon Dots (CDs)

Carbon dots (CDs) are generated using thermal evaporation, microwave, electrochemistry, micro plasma, and advanced oxidation processes techniques. The quality of CDs made by various procedures varies [144]. The most popular approach for CDs synthesis is hydrothermal/solvothermal synthesis. To make CDs, the organic material is dispersed in water or a solvent, put into a Teflon-lined steel sterilizer, installed in a microwave, and heated to a specific temperature for a predetermined time.

This method has several advantages, including a straightforward procedure and operation, consistent CDs formulation with maximum yield, and the convenience of inserting other elements and altering CDs variety [145, 146]. CDs have a high concentration of pollutants due to their substantial effective surface area, consistency, and richness of functional groups. Hence, they garner much attention in pollution control [147].

#### 8.1.2. Carbon Nanofibers (CNFs) and Carbon Nano-Tube Fibers (CNTs)

In the 1960s, carbon nanofibers (CNFs) became the most significant industrial materials in science and technology. CNFs were made from carbon precursors using melt spinning techniques. Polyacrylonitrile (PAN) was used as the primary precursor for various changes, including adding additives, oxidation stabilization at relatively low-temperature, and extending during stabilization, and carbonization. A catalytic vapor deposition (CVD) approach created vapor-produced carbon fibers (VGCFs). CNTs are narrow ducts composed of uniform carbon layers revealed amid VGCFs. The technique of formation was claimed to be arc-discharging. Carbo nanotubes (CNTs) are critical materials for nanotechnology advancement in the twenty-first century [148]. CVD is used to make CNFs by catalyzing the breakdown of carbon-containing gas with metal particles. Acetylene, ethylene, methane, and propylene are all common gases. When utilized by dissolved KOH, the total area of the CNFs increased by 4–7 times [149].

Arc discharge uses a locked reaction chamber filled with H_2_, He, and other gases. The cathode is a thick carbon rod, and the anode is supplied with Fe, Co, Ni, and other metal catalysts, with electricity created by the spark seen between graphite electrodes. The inside wall of the reaction vessel can be used to produce high-purity SWCNTs. CNTs with higher production, excellent stability, and a short preparation time are produced using the arc discharge method. The catalyst can be modified to alter the layers of CNTs. The arc discharge is similar to laser evaporation in terms of technique. In an elevated graphite tube, a sensor disperses the metal oxides catalyst and graphite nanocomposite rod. The product is accumulated on a water-cooled copper beam by moving inert gas. This technology is unsuitable for large-scale production due to high energy utilization, preparation costs, and difficult popularization. To produce carbon nanotubes, CVD is being used to catalyze the elevated degradation of ethanol, ethylene, ethane, and some other hydrogen compounds [147].

#### 8.1.3. Carbon Nanomaterials with a High Dimensionality

Graphite nanosheets are composed of a single hexagonal arrangement of carbon molecules, each covalently connected to three others. In contrast, graphite comprises various graphene nanosheets connected using van der Waals bonds [150]. In 2004, Geim and Novoselov won the award for their efforts in creating graphene from duct tape and graphite. Since its discovery, graphene research has surged. Graphene is a superconducting material that is extraordinarily strong [147]. It has significant benefits for water treatment because of its thinness, low weight, photoactivity, huge surface region, and chemical resistance. Graphene has aroused the interest of a broad spectrum of applications due to these advantages [151].

Chemical methods are used to functionalize graphene for environmental applications, including pure chemical processes (chemical oxidation and deposition) and extended chemical processes (electrochemistry, sol-gel, microemulsion, and hydrothermal procedures). These techniques can be used to introduce functional groups to carbon nanostructures for various purposes [152].

## 9. Nano-Based Bioremediation and Biotransformation

Industrial wastewaters, spillages on land and water, and improper disposal of toxic elements and their compounds are all sources of heavy metal poisoning. Despite heavy metal accumulation in the food web because of their absorbent qualities, metals are ingested by plants and animals. Heavy metals are extremely toxic and can cause various issues in animals and plants, including genetic problems. Several techniques for assessing toxic metals at incredibly low ppm and ppb concentrations have been developed. Nanoparticles are being utilized to remove toxic metals from the soil, which leaves a lot of space for R and D. Even nanoparticles adsorb on biomaterials, resulting in bio-nano composites used in wastewater treatment. Nanocomposites' adsorbent capacity has had a significant impact on the treatment of soil contaminants. The removal of heavy metals has been demonstrated with chemical-based magnetic nanoparticles. Metal ions immobilization or in situ clean-up can be aided by zero-valent iron oxide nanoparticles. Nanomaterial-based optoelectronic instruments for toxic metals identification are being developed in addition to reducing metal toxicity. The precision, accuracy, and robustness of nanomaterials make them ideal for heavy metal detection and bioremediation. Fluorescence-based transference (FRET)-based instruments also have opened up new possibilities for detecting specific heavy metals that represent harm to the environment sensitively and quantitatively. These nanoparticles contribute to sustainable development because of their wide variety of applications and efficiency [153].

To keep pest populations at bay, pesticides are utilized. They're divided into groups based on where they came from and what kind of insect they're after. Chemical pesticides are commonly employed in agricultural fields. Furthermore, excessive usage of these substances has detrimental consequences for ecological environments, such as lowering pests' pollinator numbers and posing a risk to endangered animals and birds. To reduce insecticide levels in the soil, many techniques due to surface adsorption, ultrafiltration, and microbiological destruction have been developed continuously. However, these methods have several shortcomings, such as a lack of specificity and sensitivity, as well as a long response time. As a result, nanotechnology has emerged as a valuable pesticide clean-up and detection [154].

Long-term in vivo investigations in mice indicated that DMSA-coated nanomaterial accumulated in the spleen, liver, and lung tissues for three months without causing harm. During this time, nanoparticles undergo biotransformation, which might involve size reduction, particle aggregation, or both. The research was done, including the changes of magnetic nanoparticles administered at low concentrations using a rat model. Animals were given particles with two different coatings to investigate the role of coating in particle degradation. According to the observations, animals quickly assimilate low amounts of magnetic nanoparticles. NP-DMSA was not detectable in the extravascular space 24 hours after treatment, despite a nanomaterial concentration four times cheaper than in previous trials. In comparison to control rats, animals given a low-dose of NP-DMSA had a higher level of ferritin, an iron storage protein, in their liver tissues, showing that the particles were quickly metabolized into ferritin iron. On either hand, it was observed that 24 hours after a low-dose therapy in many organs. Because of the NP-extended PEG-(NH_2_)_2_'s circulation periods, particles' arrival in the tissue is likely delayed; that is why the granules hours after delivery might be noticed. The PEG coating may help protect the nanoparticles from the reticuloendothelial system's fast breakdown. Knowledge of biodistribution, dissolution rate, and disintegration processes is essential to a better understanding of the monitoring of this variety of nanocomposites for biomedical applications [155].

## 10. Detection of Environmental Pollutants by Nano-Enzymes

Synthetic enzymes, also known as nano-enzymes, have received much attention in recent times due to their superior enzyme-like qualities to natural enzymes. Transition metals' inherent enzyme activity is being studied for biomaterials, bactericidal, cytoprotecting, anti-cancer, tissue regeneration, and drug delivery, among other applications [156].

Because of their excellent physical and chemical qualities, cheap expense, high stability, and simplicity of storage, nano-enzymes have improved dramatically in the recent decade. They can be used as a link to natural enzymes. These are enzyme-like nanomaterials similar to natural enzymes in the percentage of overall size, configuration, and surface charge. In biomedicine, nano-enzymes have shown promise in anti-bacterial drugs, bio-detection, and treatment for cancer [157].

It has been investigated whether microbial-derivednano-enzymes may be used to treat two important environmental pollutants potentially. Polyaromatic hydrocarbons (PAHs) are among the most significant industrial contaminants due to their extensive presence, toxicity, and proclivity for bioaccumulation. Furthermore, bioaccumulation caused by bacteria organisms has significant environmental and economic implications in many industry sectors, particularly maritime vessels, where it increases hydrodynamic drag due to loss of rotational speeds at constant potential or a power significantly improve to retain the same intensity, resulting in greater emissions and energy, particularly greenhouse effect carbon dioxide, into the environment (GHGs). Among the clean-up alternatives, biological routes have been considered promising, efficient, and long-term. Organic ligninolytic enzymes are perfect for PAH breakdown and antifouling. However, due to many reasons, including their limited stability, flexibility, and high manufacturing cost, these enzymes are difficult to use on a big scale. In recent years, it has been shown that nanoparticles, particularly nano-enzymes, are a new and synergistic technique for detoxifying contaminated areas while retaining enzyme consistency [158].

Transition metals' intrinsic enzyme activity is being investigated for biomaterials, bactericidal, cytoprotecting, anticancer, tissue regeneration, and drug delivery, among other applications. Silver (Ag) is one of the transition metal oxides investigated significantly since the turn of the century due to its biological activity and catalytic properties. In the form of metal-organic systems, complexes, bimetallic, and hexacyanoferrate structures, the authors compiled a list of Ag-based hybrid enzymes. According to the literature, Ag-based hybrid enzymes can detect various compounds, ions, and biological species. Furthermore, antimicrobial, cell defense, and anticancer applications are being researched using Ag-based enzymes. Hybrid enzymes based on Ag have a lot of potential for clinical trials and commercialization [159].

## 11. Mechanisms of Nano-Based Wastewater Treatment

There are several conventional adopted treatment methods available for water pollutants, such as coagulation, flocculation, reverse osmosis, flocculation, and filtration. Still, these are generally not that efficient in removing all the target pollutants. These have gradually increased the need for resilient techniques and membranes that can be used for water treatment and its purification [160].

The application of nanomaterials was investigated to develop separation media with great supremacy in respect of responsiveness or output to refine traditional treatment techniques [161]. Many treatment facilities have implemented nanofiltration to produce an effluent with minimum pollution levels [162]. Nanomaterials have also been recognized for their usage in sterilizing water and bio-remediating wastewater [161, 162].

### 11.1. Nano-Based Membranes

**Figure 3:**
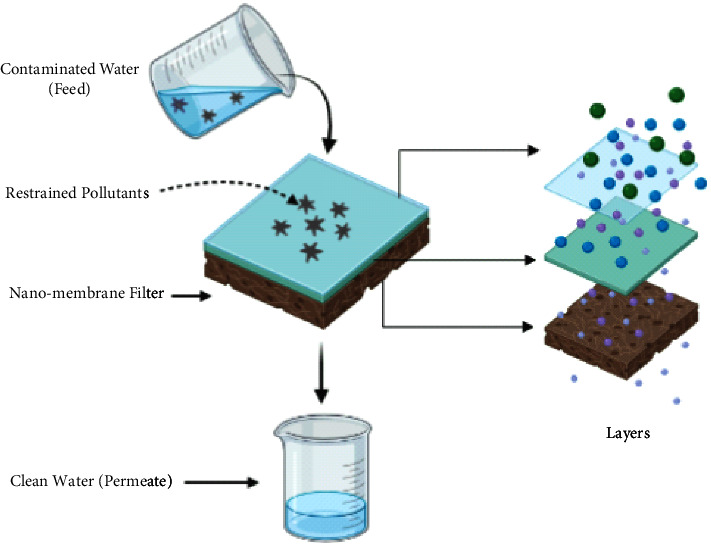
Mechanism showing nano-membrane filtration.

Membrane filtration has an influential role in remediating water from various pollutants. In the last decade, the development of ceramic and polymeric membranes has positively affected the use of membranes. However, membrane fouling is considered one of the significant issues in the filtration process that raises serious concerns about the viability of membrane use. Nanotechnology is quite useful in the fabrication of water purifying membranes. A recent study reported the fabrication of water filtration nanostructured membranes using nanomaterials such as carbon nanotubes and nano-reactive membranes [163]. A generalized schematic diagram for nanomembrane filtration is shown, which retains specific pollutants by using coating technology, decomposing contaminants, and photocatalytic oxidation [164] (Figure 3).

Similarly, for efficient viral separation, the nanostructure surface changes microporous ceramics [165]. A colloidal nano-dispersion of hydrated yttrium oxide was used to coat the interior surface region of the device. It was also heat-treated to give it an electropositive Y_2_O_3_ coating. The modified nanostructure filters successfully removed 99.9% of MS2 bacteriophages with a diameter of 25 nanometres from feed water with a pH of 5–9. Furthermore, membranes for water treatment that are made of nano-reactive material are synthesized. In an aqueous solution, these membranes degrade contaminants like 4-nitrophenol [166] and bind metal ions [167]. Silver nanoparticle-loaded polysulfonate UF membranes were effective against *E. coli* K12 and *P. mendocina* bacteria strains and improved virus removal significantly [168].

Organic contaminants going through simultaneous filtration and photocatalytic oxidation were achieved by fabricating TiO_2_ nanowire membranes. Another work used extrusion and sol-gel/slip casting to create TiO_2_/Al_2_O_3_ composite membranes that successfully degraded Direct Black168 dye [169].

### 11.2. Nano-Adsorbents

**Figure 4:**
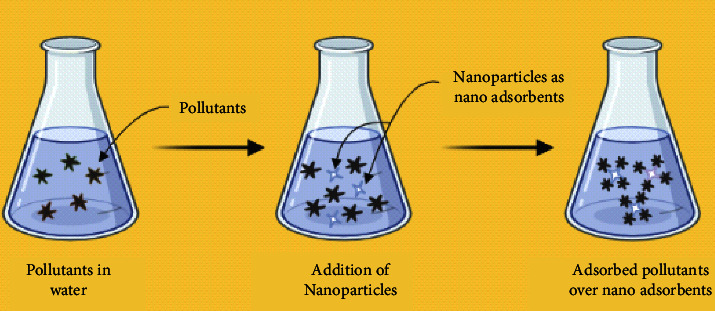
Mechanism of nano-adsorbent in wastewater treatment [173].

Nowadays, as the hunt for low-cost adsorbents with improved metal-binding capacities has intensified, adsorption has become one of the surrogate treatment methods [170]. Many different materials are now employed as sorption sites in nanoparticles and as separation media for heavy metals like Cd^2+^ that are arrested using nitric acid, hydrogen peroxide, and other chemicals [171]. These are oxidized to form a high adsorption capacity for metal ions and quicker kinetics in association with various functional groups such as hydroxyl and carbonyls [172]. A generalized schematic diagram for the nano-adsorption mechanism is depicted (Figure 4). This operation is based on two significant steps. Nanoparticles that operate as adsorbents are permitted to mingle with polluted water flows in the very first phase. As a result of their great adsorptive power, these applied nanoparticles arrest any pollutants nearby in the last stage. This is how nanoparticles adsorb all of the contaminants present in water.

Many research on the use of nano-adsorbents in the removal of harmful elements in ionic forms, such as As, Cr, Cu, Pb, and Ni, are accessible in the literature [174]. The use of nano-adsorbents in removing hazardous metals such as As, Cr, Cu, Pb, and Ni in ionic forms has been studied extensively in the literature. Magnetite nanorods can be extensively used to remove Fe^2+^, Pb^2+^, Cd^2+^, and Cu^2+^. In some circumstances, nanorods outperform nanotubes in terms of adsorption capacity, for example, in the removal of Zn^+2^ and Pb^2+^, but not in the removal of Cu^+2^. Other forms include super-paramagnetic nanoparticles that bind to Hg^+2^ more quickly and selectively [175].

## 12. Natural Compound-Based Nanomaterials and Their Applications

Natural nanomaterials are created by physiological and biochemical functions in the earth, ocean, climate, and air. Natural nanomaterials can be found in the atmosphere [176]. Weathering and mineral deposition in soils provide the majority of natural nanomaterials in the atmosphere's critical zone [177].

Clays are the most common inorganic nanomaterials found in nature. Other weathered natural nanomaterials exist at lower concentrations than clays but play essential biogeochemical functions [178]. All living creatures produce/secrete nanoscale biomolecules such as immune cells, enzymes, and matrix proteins composed of Langmuir Blodget films (3–5 nanometres). Other nanometre-sized substances released in the body are also necessary for the human body's normal functioning [178].

Bone is a 100–200 nanometre-diameter open-cell material composed of protein filaments and collagen fibrils: calcium phosphate crystals, and a complex vascular system (35–60 nm in diameter). Bone organizes into a five-level structure at the macroscopic scales [179, 180].

Inorganic minerals (primarily calcium and phosphate-containing hydroxyapatite crystals, and trace amounts of sodium, potassium, magnesium, fluorine, chlorine, and some trace elements such as silicon, strontium, iron, zinc, and copper also help to maintain and build bones [181].

Nanostructures have also been discovered in insects, connected to many physical and physiological functions. Friction, chemical sensing and responsiveness, visual acuity and management, movement, cell interaction, and temperature regulation are among the functions outlined [182, 183].

Birds use their elaborate colored feather patches and unique traits to find a mate, defend themselves from predators, and differentiate themselves from some other birds within the same species [184]. Plant natural fibers are nanometre hierarchical bio-composites of cellulose fibrils found within the cells. The shortest cellulose fibrils are 100–1000 nm long and have crystallinity segments [185].

## 13. Metal-Based Nanomaterials for Photocatalytic Process

Photocatalysis is a set of chemical reactions triggered by electromagnetic light [186]. Nanomaterials have become one of the most critical influences on technology and biomedicine. To make use of the unique features of nanomaterials, the photocatalytic method has been used in various applications, including the removal of pollutants from the water and air. This technique is also being utilized to generate power.

### 13.1. Mechanism of Photocatalysis

**Figure 5:**
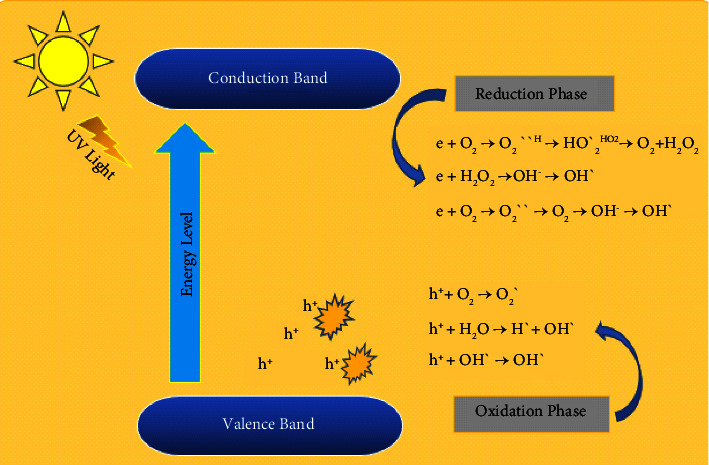
Basic mechanism of photocatalysis [190].

The entire basic process of photocatalysis is done in two phases named reduction phase and another one is oxidation phase [187] (Figure 5). When photons illuminate a matter with an energy that is the same or greater than the matter's bandgap, its electrons jump from the conduction band (CB) to the valence band (VB) through the bandgap exiting positive hole pairs. This phase is called the reduction phase. After the reduction phase, these electrons and hole pairs build reactive oxygen species (ROS) like O_2_ and OH. This is the oxidation phase. The sort of reactive oxygen species depends on different matters and illuminating photons. The construction of reactive oxygen species is excellent in photocatalysis because it exerts various activities such as dye degradation [188] and antibacterial effect [189].

The use of semiconductor materials as a photocatalytic medium to eliminate the organic and inorganic species has piqued attention in recent years. Because of its high oxidation capability, this approach has been proposed as a powerful tool for environmental conservation [191]. Nanomaterials for example oxides [192–194], semiconductors [195, 196], metals [197, 198], and graphene [199, 200] have proved to have a significant impact on photocatalysis processes [201, 202] because of their increased and tunable optical characteristics, they are effective photocatalysts (Table 3).

## 14. Eco-Friendly Nanotechnologies to Control Environmental Pollution

The environmental study has found nanotechnology as a key player in the evolvement of recent environmental engineering and scientific approaches. The nanomaterials and polymer nanocomposites are the topics of a review of the current status of devices based on nanotechnology, which show significant outputs in environmental studies. Nanotechnology has substantially contributed to environmental restoration and conservation by tackling long-term human health threats. Many nanostructures have been developed in this field, and more are in development. Without a doubt, the environmental field has provided us with numerous benefits and performed a significant role by using fewer amount chemicals, preserving power, reducing wastages, improving atom's proficiency, and building more advanced technology and components for the remedy of environment, all of which promise ecological integrity [204]. One of the most severe worldwide food and environmental risks is contamination by artificial chemicals and substances in nature. Heavy metal wastages like pesticides, insecticides, and herbicides from industrial androgenic processes are being used excessively in agriculture, and pharmaceutical overuse of antibiotics, and medications [205, 206]. Water conservation is one of the essential resources for human existence and other living forms on the planet's survival and future improvement. With the increasing population growth, technological advancements, urbanization, and industrial expansion, demand for fresh water is also rising [207]. One of the primary reasons for water contamination is because of those heavy metals that are generated from nuclear power plants, mining, chemical treatment plants, metallurgy, electroplating, and home-based and agricultural effluents [208–211]. The food chains are contaminated with the aggregation of heavy metals such as Cu, Pb, Zn, and Hg and as a result, it causes critical long-term health-associated risks to all [212–214]. The microbial operation cannot degrade heavy metals as they are carcinogenic and poisonous, affect the ecosystem and environment, and also build up in the food chain [215]. Compared with other current approaches, adsorption methods are more acceptable as they are simple, cost-effective, eco-friendly, have enough practicability and easy regeneration of sorbents for the elimination of heavy metal pollutants [216–218]. The table below (Table 4) shows the recent discovery of numerous nanoparticles used for heavy metal remediation.

## 15. Nanomaterials and Their Applications to the Environmental Pollution

Nanoparticles are highly reactive as they have active sites and an enormous surface area to the ratio of volume. Nanoparticles' features can be used to solve prospective environmental challenges such as air, water, and soil contamination and their recovery. A variety of nanomaterials could be used to improve the environment's health and the lives of the earth's creatures.

### 15.1. Nanomaterials for Environmental Contaminant Detection

Contaminants and harmful pollutants are commonly found in water, soil, and air in combination. Therefore, this kind of equipment is rare that can filter, sense, and monitor toxic particles from the soil, water, and air. In this aspect, nanotechnology has a wide range of solution options to solve these issues. Nanomaterials can perform as excellent adsorbents, catalysts, and detectors [241]. Industries are using nanosensors (nanomaterials and polymer composites) to trace different pollutants, including metal pollutants, organic and inorganic pollutants, dangerous gases, and biological elements. Numerous hazardous gases in our environment can create significant health problems and environmental damage such as air pollution and water contamination. Nanosensors make it much easier to identify harmful environmental chemicals and heavy metals in the environment [242]. Various nanosensors can be used to detect such toxic gases (Table 5).

### 15.2. Nanoparticles for the Purification of Air

The air pollution rate is rising day-by-day due to people's high standard of living has been a severe problem. This also expedites the way of world overheating. Nanotechnology has discovered several methods for reducing air pollution. Carbon nanotubes and gold nanoparticles are the most important uses of nanotechnology as they adsorb poisonous and dangerous gases in the airspace. Single-walled nanotubes (SWNTs) and multiwalled nanotubes (MWNTs) are one-dimensional macroparticles with exceptional thermal stability and chemical properties that show tremendous efficacy as excellent adsorbents for removing different pollutants, including inorganic and organic [245]. Using nano-catalysts, contaminants can be transformed quickly and selectively [24]. The use of nanofillers is another way of improving air quality. Porous membranes with pores small enough to segregate different contaminants are used in this method. Filters made on metal oxide frameworks (MOFs) have been proven effective in the filtration of particulate particles.  Table 6 shows a list of nanoparticles that control air pollutants [246, 247].

### 15.3. Nanomaterials for Water Treatment

Globalization and industrialization are the main reasons for the current levels of deadly pollutants and toxins found in the water. There are several examples of water pollution like industrial wastage, oil spills, herbicides, and pesticides, fertilizer leakage, industrial by-products, and the exploitation of fossil fuels. Nanotechnology is being developed to improve water quality and a reactive channel for bioremediation, separation, and filtration, and disinfection [14]. Nanomaterials have excellent advantages like large exterior space for better reaction at a small corelative weight. They are more cost-effective than activated carbon and also have the potential to remove pollutants [248].

For pollution management, Fe-nanoparticles could be inserted directly into the soil, sediment, or solid waste. Nanoparticles are combined with water to create a slurry in this process. The nanoparticles are injected and remain suspended, resulting in the formation of a therapy zone. Activated carbon is the example of another approach, which is quite adequate, is to capture the nanomaterials with a solid matrix or exterior. Other metals like Zn and Sn, which can decrease pollutants such as iron, could be substituted for iron nanomaterials [249].

**Figure 6:**
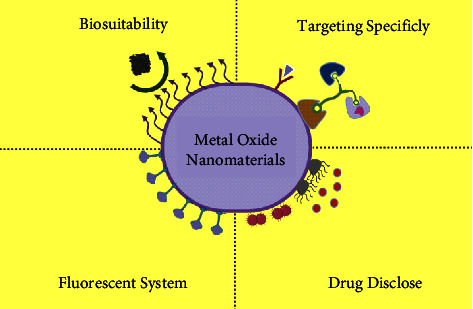
Various use of metal oxide nanoparticles [251].

Metal oxide nanomaterials have shown great promise as an economical, eco-friendly, and long-term wastewater management solution (Figure 6). These nanoparticles are contractible without significantly reducing surface area, have a large specific surface area, a short intraparticle diffusion gap and have far more attachment sites. Also, they are simple to recycle. Furthermore, few of them have higher adsorption capability to activate carbon for their superparamagnetic properties [250].

Adsorption is mainly accomplished through complex formation between oxygen molecules and dissolved metals in metal oxide groups. It is commonly recognized that rate-limiting intraparticle diffusion triggers effective sorption of heavy metal on the outer layer of metal nanoparticles along the borders. The creation of novel sorption sites on the magnetite surface has already been attributed to the alteration of nanoparticles on the surface. Al, Zn, Ti, and Fe metal oxides are inexpensive and excellent adsorbents for toxic substances and radioactive elements [252].

As a photocatalyst oxide, TiO_2_ is used in sophisticated photochemical oxidation methods to treat water for organic and inorganic contaminants. The surface of TiO_2_ that has been transformed into nanotubes is more effective at converting organic and inorganic pollutants. TiO_2_ electrodes are particularly successful at determining the chemical oxygen requirement of water and can thus be used as sensors to identify contaminants in water [253, 254].

### 15.4. Nanoparticles for Land Waste Erosion

**Figure 7:**
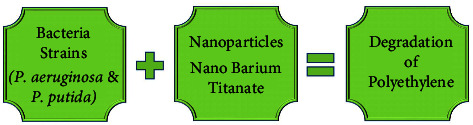
Mechanism of nanoparticles-base polyethylene degradation using microorganisms [255].

The growing standard of living has resulted in a slew of significant issues, one of which is the accumulation of land waste, which necessitates innovative solutions and technologies. Polythene and plastic are the most difficult to degrade all industrial and residential land waste, and disposing of them in the junkyard is also not a proper solution. More than twenty-five million pieces of plastic garbage are gathered worldwide. Many microorganisms, such as *Pseudomonas aeruginosa* and pseudomonas putida, have been fruitful at deteriorating polythene (Figure 7).

By increasing the growth phase of polythene degrading bacteria, nanotechnology aids in the degradation of these terrestrial pollutants. Nano barium titanate, super-magnetic Fe-oxide and fullerene (60) nanomaterials are among the bacteria growth-promoting nanomaterials (SPION). In the case of low-density polyethylene (LDPE) degradation, TiO_2_ dope with different metals is an example (Fe, Ag, or Ag + Fe). This method also shows significant activity because of the photocatalytic activity of TiO_2_ that is initiated by UV radiation [256].

## 16. Recent Advantages and Prospects

The mean particle size, the manner of clumping, the size of particles, the statement of the particle number size distribution surface area, and structure are all essential characteristics of nanomaterials. Nanomaterial is an emerging interdisciplinary topic of research concerned with developing various ways to manufacture nanoscopic bits of the desired material, such as polymers, semiconductors, and other materials.

Photocatalysis has emerged as a promising and creative technology that has the potential of transforming solar energy into chemical energy [257]. Typical inflictions of the photocatalysis method include water disinfection, photoelectric detection, photodegradation of pollution, and heavy metals, hydrogen generation, CO_2_ photoreduction, and photodynamic therapy [258, 259]. For photoelectric sensing, various oxides have been utilized, including ZnO, TiO_2_, SnO_2_, and WO_3_ [51]. In the case of photoelectric sensing, TiO_2_ was the most promising of these oxides used to track the appearance of several gases in a given field [260].

Recently, Wu used nitrogen ion implantation to develop the visible light photo-response of a single TiO_2_ nanowire and for the first-time, visible light detection was done by applying the N-doped single TiO_2_ nanowire. The photoelectric characteristics were increased with a high-level electrical field (bias voltage- 3V) and the same strength of light irradiation, resulting in more charge carriers being activated. As a result, reconnection of the electron-hole pairs is tougher with a higher bias voltage. Therefore, the expanded photodetector, based on a single N-TiO_2_ nanowire, performs admirably in visible light detection, with a responsivity of 300 AW1 at a bias voltage of 3 Volt [261].

Nickel oxide (NiO) NPs are researched widely because of their electrocatalysis, high chemical stability, super conductance properties, and electron transfer capabilities [262]. NiO has a wide range of possible applications like electrochemical performance, water treatment, antibacterial properties, and gas sensing. Several ways for producing NiO NPs have been documented, including solvothermal [263], precipitation-calcination [264], chemical precipitation [265], microwave-assisted hydrothermal [266], and thermal decomposition [263, 267] methods.

Nanotechnology allows scientists to generate environmentally beneficial compounds or materials that can easily replace harmful materials when applied. Specific biocompatible nanomaterials surrounded by definite metal ions could be successfully employed in contaminant treatment technologies that use ideas of environmental nanoscience, depending on the nature and complexity of the pollutants. The following are potential prospective nanotechnology trends, as well as some intriguing new developments and uses- nanomaterials have contributed significantly to wastewater treatment, and they continue to have the capability to take about new creative techniques for the treatment of wastewater. For instance, in wastewater treatment membrane outpouring, electrospun nanofibrous membranes (ENMs) have already been applied broadly. Despite the high cost, they utilize polymers together because of outstanding hydrophobicity polytetrafluoroethylene (PTFE) and polyvinylidene fluoride (PVDF). Consequently, for membrane distillation application of wastewater treatment, polyethylene terephthalate (PET), like alternative polymers, should be electrospun into ENMs [268].

For wastewater treatment, polymer nanocomposites (PNCs) are also another exciting application of nanotechnology that can be improved. PNCs have the constraint of being able to execute only one function. As a result, researchers should develop and deploy multifunctional PNCs that will improve PNCs' water remediation capabilities [267]. Many diseases have been linked to air pollution. The promise for air purification has already been elucidated by several nanotechnology applications like nanoparticles, nanomembranes, and nanofillers. These inflictions might broaden in the future. For instance, in photocatalysis or chemical catalysis decomposition in air purification, polymer-assisted nanocomposites should be extensively accepted. Several surface modification processes are being used to test different air adsorbents such as TiO_2_, polymer sorbents, and other nanomaterials for increased selectivity [241].

The scope of nano air purification can be expanded in the future to include a variety of particulate matter filters. While researching the current R&D situations, it is possible to introduce nanomembranes for automobile silencers, air conditioners, freezers, fume pipes, and other applications that can filter and diffuse dangerous particles before they are released into the environment.

## 17. Conclusion

Environmental pollution poses a massive threat to the entire living community. Several manufacturing activities such as the emission of industrial chemicals, excessive burning of fossil fuels, exhausting fumes from automobiles, noneco-friendly technologies, construction and demolition, pull-down of wild forests, and other factors contribute to the rapid increase in environmental pollution. To avoid this degeneration, concrete and long-term efforts should be made. In recent decades, developments in nanomaterials and nanotechnologies have benefited environmental detection and remediation. This manuscript discusses various types of synthesis, characterization, different utilization, and scopes of bioinorganic-based nanomaterials for environmental pollution. Integrating nanomaterials with analytical techniques has a lot of appeal for developing simple, sensitive, low-cost, and miniaturized devices for detecting a range of environmental pollutants in the field. Nanotechnology has an extensive length of implementations in all disciplines of technicality. Spite fullest, still, there is much more to discover. Many attempts are going on to advance nanotechnology and remove its drawbacks, including toxicity. Nanoparticles, which can be active on small and big scales, can provide a more sustainable, cost-effective, and holistic strategy for addressing this serious problem.

## Figures and Tables

**Table 1:** Microorganism-based synthesis of diverse nanoparticles.

Sr. no	Microorganisms	Products	Shape	Location	Reference
1	*A. Flavus*	Ag	Spherical	Extracellular	[95]
2	*A. Fumigatus*	Ag	Spherical	Intracellular	[96]
3	*Bacillus Cereus*	Ag	Spherical	Extracellular	[97]
4	*C. glutamicum*	Ag	Irregular	Extracellular	[98]
5	*D. desulfuricans*	Pd	Spherical	Extracellular	[99]
6	*Enterobacter spp.*	Hg	Spherical	Intracellular	[100]
7	*Escherichia Coli*	Au	Triangles, hexagons	Extracellular	[101]
8	*Fusarium oxysporum*	Pt	Spherical	Extracellular	[102]
9	*Plectonema Boryanum*	Au	Cubic	Intracellular	[103]
10	*Rhodococcus spp.*	Au	Spherical	Intracellular	[104]
11	Yeast	Au/Ag	Irregular	Extracellular	[105]

**Table 2:** Different nanoparticles with their fundamental characteristics.

Sl. No	Nanoparticles	Characteristics	Reference
01	Aluminum	High reactivity, sensitive to moisture, heat, and sunlight, large surface area	[115]
02	Aluminium oxide	Increased reactivity, sensitive to moisture, heat, and sunlight, large surface area	[116]
03	Iron	Reactive and unstable, sensitive to air (oxygen), and water	[117]
04	Iron oxide	Reactive and unstable	[118]
05	Silver	Absorbs and scatters light, stable, anti-bacterial, disinfectant	[84]
06	Gold	Interactive with visible light, reactive	[119]
07	Cobalt	Unstable, magnetic, toxic, absorbs microwaves, magnetic	[120]
08	Cadmium	The semiconductor of electricity, insoluble	[121]
09	Lead	High toxicity, reactive, highly stable	[122]
10	Copper	Ductile, very high thermal and electrical conductivity, highly flammable solids	[123]
11	Zinc	Antibacterial, anti-corrosive, antifungal, UV filtering	[124]
12	Zinc oxide	Antibacterial, anticorrosive, antifungal, and UV filtering	[125]
13	Titanium oxide	High surface area, magnetic, inhibits bacterial growth	[126]
14	Magnetite	Magnetic, highly reactive	[127]
15	Silicon dioxide	Stable, less toxic, able to be functionalize many molecules	[128]
16	Cerium oxide	Antioxidant, low reduction potential	[129]

**Table 3:** A list of metal-based nanomaterials that are being used in the photocatalytic process.

Sr. no	Nanoparticles	Irradiation	Mechanism of photocatalysis increment	Reference
01	TiO_2_/WO_3_	Sunlight/solar UV-visible light	Heterogeneous	[203]
02	ZnO/Ag	UV-(320–400 nm)	Heterogeneous/SPR	[185]
03	WO_3_/TiO_2_	UV-(365 ± 5 nm)	Hetero-junctional electrical flaky method	[195]
04	p type-FeHO_2_/n-type WO_3_ H_2_OZn and Cu doped WO_3_	UVA (365 nm)Visible light-(>420 nm)	Semiconductors-heterogeneous	[200, 201]
05	N-TiO_2_	Visible-light	Doping method	[202]
06	Fe - doped response TiO_2_ films	Visible-light	Doping method	[202]
07	TiO_2_/GO composite nanowires	Visible-light	Heterogeneous	[197]
08	Au-doped PdO nanomaterials	Ultra-violet (254 nm)	Ultra-violet + H_2_O_2_-system batch system	[188]
09	Silver nanomaterials on Cadmium-II boron imidazolate	Ultra-violet	SPR method	[187]
10	Ag-polymer core-shell nanomaterials	Ultra-violet A	SPR method	[195]

**Table 4:** Recent findings of nanoparticles that makeover heavy metals.

List of adsorbents	Heavy metals	Adsorption capacity (mg/g)	Reference
*DES-*functionalized carbon nanotubes	Hg^2+^	186.93	[219]
Amino-functionalized Fe_3_O_4_ (iron oxide)/MWCNTs	Cu^2+^	30.5	[220]
Functionalized GO (graphene oxide) CA beads	Pb^2+^	601	[221]
Si-nanospheres	Cu^2+^	139.7	[222]
Si-phy-(nano-polyaniline) NPANI	Pb^2+^	186.1	[223]
Hematite magnetic nanomaterials	Cr^6+^, Cu^2+^, and Pb^2+^	201, 34.2, 68.8	[224, 225]
Amino-functionalized Fe_3_O_4_ nanomaterials	Ni^2+^, Cr^6+^	222.15, 232.50	[226]
*PTMT* (organo-disulfide polymer)/Fe_3_O_4_	Hg^2+^, Cd^2+^, and Pb^2+^	603.2, 216.6,533.2	[227]
Hydrous ferric oxide (HFO)-CMC-NC	As^5+^	355.1	[228]
MnO_2_/gelatin	Cd^2+^, Pb^2+^	105.2, 318.8	[229]
Casein-coated ZnO nanomaterials	Cd^2+^, Co^2+^ and Pd^2+^	156.8, 67.83,194.97	[230]
TiO_2_-chitosan nanomaterials	Cd^2+^	1800 micro mole/g	[231]
Al_2_O_3_ (aluminum oxide) nanomaterials	Pb^2+^, Cd^2+^	47.1, 17.3	[232]
MgO (magnesium oxide) nanomaterials	Cd^2+^, Pb^2+^	2293, 2615	[233]
CeO_2_ (cerium oxide) nanomaterials	Pb^2+^, As^3+^	23.2, 71.8	[234, 235]
CNTs/PAMAM (poly-amidoamine dendrimer) NPs	As^3+^, Zn^2+^, Co^2+^	433, 471, 495	[236]
ZnO/chitosan nanomaterials	Cd^2+^	135.2	[237]
Alginate/chitosan nanomaterials	Cr^6+^	108.6	[238]
Fe_3_O_4_ (iron oxide)-SiO_2_/Zr-MOFs (metal-organic frameworks)	Pb^2+^	102.1	[239]
Fe/MgO NPs	Pb^2+^	1476.5	[240]

**Table 5:** Numerous nanoparticles for various analyte detection [243, 244].

Serial no	Name of nanoparticles	Detection of analyte
01	ZnO nanofibers/reduced GO	NH_3_
02	Au nanoparticles, carbon or SPCEs + Au NMs, paper electrodes with carbon ink + carbon NMs + Au NMs	Hg-II in water
03	WO_3_ + graphitic *C. Composites* nitride (g-C_3_N_4_)	Ethanol gas
04	Single-walled carbon nano-tubes-Sn/SnO_2_ nanocomposite	Ethanol molecules
05	Pb NMs on *C. pasteurianum* (bio-nano met)	Methyl-orange and evan's blue (azo dyes)
06	Biopolymer-based carboxymethyl cellulose - stabilized (Fe-NPs)	Cr (VI) toxicity
07	Iron-oxide and Cu NPs	As-III, As-IV ions, and Pb-II serially
08	Ag NPs + Cu NPs + CNTs/chitosan multifunctional composites	Cu-II, Cd-II, and Pb-II
09	Metal-doped TiO_2_	H_2_O pollutants like 2-chlorophenol, *E. Coli*
	Titrate nanotubes	Gaseous NO
	SWCNTs/QDs/antimony NMs	Detection of Cd-II

**Table 6:** Various nanomaterials to destroy, uncover, and minimize air pollutants.

Serial No	Nanomaterials for the sanctification of air pollutants	Uncovered contaminants
01	Metal-organic frameworks-based nanowire filters	*E. Coli* destruction; CO_2_ level reduction; PM (particulate matter) removal
02	Gold-nickel oxide core-shell nanomaterials-(p-type gas sensor)	C_2_H_5_OH at (200°C)
03	Copper-copper oxide NPs	Reduction of O_2_
04	God-titanium oxide NPs on SiO_2_ matrix	NO oxidation to NO_3_^_^ and NO_2_^_^ (lowering the quantity of NO_2_ released)
05	Silicene (allotrope of sicon)	SO_2_ detection
06	TiO_2_-coated pleated washable synthetic (PWS) fiber	Oxidation (photocatalytic) of VOCs and CO

## Data Availability

All available data used to support the findings of this study are presented in the manuscript.
